# Muscarinic Cholinoreceptors in Skeletal Muscle: Localization and Functional Role

**DOI:** 10.32607/actanaturae.25259

**Published:** 2023

**Authors:** I. V. Kovyazina, A. A. Khamidullina

**Affiliations:** Kazan State Medical University, Kazan, 420012 Russian Federation; Kazan Institute of Biochemistry and Biophysics, FRC Kazan Scientific Center of RAS, Kazan, 420111 Russian Federation

**Keywords:** skeletal muscle, neuromuscular junction, acetylcholine, muscarinic cholinoreceptor, autoregulation

## Abstract

The review focuses on the modern concepts of the functions of muscarinic cholinoreceptors in skeletal muscles, particularly, in neuromuscular contacts, and that of the signaling pathways associated with the activation of various subtypes of muscarinic receptors in the skeletal muscles of cold-blooded and warm-blooded animals. Despite the long history of research into the involvement of muscarinic receptors in the modulation of neuromuscular transmission, many aspects of such regulation and the associated intracellular mechanisms remain unclear. Now it is obvious that the functions of muscarinic receptors in skeletal muscle are not limited to the autoregulation of neurosecretion from motor nerve endings but also extend to the development and morphological rearrangements of the synaptic apparatus, coordinating them with the degree of activity. The review discusses various approaches to the study of the functions of muscarinic receptors in motor synapses, as well as the problems arising when interpreting experimental data. The final part of the review is devoted to an analysis of some of the intracellular mechanisms and signaling pathways that mediate the effects of muscarinic agents on neuromuscular transmission.

## INTRODUCTION

Acetylcholine (ACh) is one of the main neurotransmitters and modulators of the nervous system. ACh receptors – nicotinic (ionotropic) and muscarinic (metabotropic) ones – are expressed in a variety of tissues, from neuromuscular junctions and the parasympathetic nervous system to the cortical areas involved in cognitive functions such as learning and memory. Cholinergic agents, including allosteric modulators, are actively used in the treatment of various pathologies [1, 2, 3].

The first studies that demonstrated the involvement of muscarinic cholinergic receptors (mAChRs) in the regulation of neuromuscular transmission go back to the 1960s [4, 5]. By now, all the five known mAChR subtypes (M1–M5) have been found in the vertebrate neuromuscular contacts, and the signaling pathways triggered by the activation of these receptors are multiple, complex, and often interrelated.

The exact location of different mAChR subtypes in skeletal muscles is not entirely clear: some of these receptors can apparently be located not only on nerve endings, but also on the sarcolemma and Schwann cells [6, 7, 8]. Multiple signaling pathways are associated with the activation of different mAChR subtypes in the vertebrate skeletal muscles: some of them alter the concentration of intracellular Ca^2+^ by regulating its release from intracellular depots or modifying the functions of the calcium channels modulating, either directly or indirectly, the neurosecretion process (e.g., via enhancement of free radical production). Other mechanisms involve direct impact on the vesicle exocytosis machinery; e.g., via the regulation of protein kinase A activity, phosphorylation of SNAP-25 protein, etc.

It is known today that the functions of mAChRs in the regulation of neuromuscular transmission are not confined to the control of neurosecretion intensity. A number of studies have revealed that these receptors are involved in the regulation of the timing of ACh release [9, 10, 11]. Muscarinic receptors, and the odd subtypes in particular, can reside on the sarcolemma and regulate the contractile activity of muscle fibers, as it has been shown for M5 mAChRs [12], or participate in the control of the resting membrane potential [13]. Relatively recently, the role of various mAChR subtypes in maintaining synaptic stability, growth, and development of motor synapses has been revealed [7]. That is, these receptors ensure the functionality of a tripartite synapse (nerve ending – muscle fiber – Schwann cell) and coordinate the development and morphological features of the synaptic machine with its activity level.

This review attempts to summarize the currently known data on the localization of mAChRs in vertebrate skeletal muscles, the effects of muscarinic agents on synaptic transmission parameters, and the signaling pathways coupled with the activation of different subtypes of mAChRs in the neuromuscular contacts.

## PHARMACOLOGICAL AND GENETIC APPROACHES TO STUDYING THE mAChR FUNCTIONS

Five mAChR subtypes (M1–M5) are distinguished depending on the localization, molecular structure, nucleotide sequence, and functions. The conserved structure of the mAChR subtypes is the reason for the poor selectivity of most of the muscarinic agonists and antagonists used for pharmacological studies and the difficulties arising when interpreting the experimental data [2, 14]. Currently, the only highly selective mAChR antagonists available are the “muscarinic toxins” isolated from the mamba venom [15].

Allosteric modulations are another pharmacological approach to the study of muscarinic functions [16]. Muscarinic receptor subtypes exhibit high structural homology in the transmembrane domains where the orthosteric binding site is located, but the extramembrane domains are less conserved. Targeted synthesis of compounds that bind specifically to the allosteric domains makes it possible to achieve a highly advantageous selectivity in binding that is otherwise impossible with orthosteric ligands [17, 18, 19].

Recently, animals with mutations in the genes encoding various subtypes of these receptors have witnessed expanded use, in addition to the pharmacological analysis, in the study of the functional role of mAChRs both in the whole organism and in individual cells. Thus, it has been found that in rats *Rattus norvegicus*, genes encoding various mAChR subtypes reside on chromosomes1 (M1 subtype), 3 (M4 and M5 subtypes), 4 (M2 subtype), and 17 (M3 subtype) [20]. These data have made it possible to develop congenic and consomic animal strains that can be used to study the functions of different mAChR subtypes [20, 21]. Research into the synaptic transmission (including in peripheral synapses) in animals with mutations in genes encoding different mAChRs have shed light on the physiological role of different mAChR subtypes. Various cognitive and behavioral abnormalities, as well as changes in the morphology of synaptic contacts and in the pharmacological effects of cholinergic agents, have been observed in animals with mutations in mAChRs, viable and fertile [1, 21, 22, 23].

## LOCALIZATION OF mAChRs IN SKELETAL MUSCLES

In the area of vertebrate neuromuscular contacts (NMJs), mAChRs can reside both on the membrane of nerve endings and the sarcolemma, as well as on Schwann cells [6, 7, 8]. These cholinoreceptors can be activated by vesicular ACh released from nerve endings either spontaneously (asynchronously) or synchronously during nervous activity, as well as by non-quantal ACh, which makes up a very significant part of the neurotransmitter in the synaptic contact area [13, 24, 25]. The presence of muscarinic receptors, and those of M1 subtype in particular, on the sarcolemma of the rat diaphragm was reported in [8]. Megan Wright et al. [7] showed that in mouse LAL muscle, M2 receptors are present exclusively in motor neurons, whereas M1, M3, and M5 mAChRs can be associated with Schwann cells and/or muscle fibers. Meanwhile, the presence of functional M1–M4 mAChRs was demonstrated by RT-PCR for a culture of Schwann cells obtained from the phrenic nerve of newborn rats, and the M2 subtype was predominant [26]. At the same time, the M4 mAChRs were expressed in the culture of Schwann cells at a very low level and the M5 subtype was not detected at all; similar results were obtained later for human Schwann cells [27].

M1–M4 mAChRs subtypes were shown to be present and functioning in the area of rat neuromuscular contact at all stages of postnatal ontogenesis [28, 29].

All the five mAChR subtypes were also discovered in the NMJs of cold-blooded animals. The presence and functional activity of M1–M5 subtypes were demonstrated by combining the immunohistochemistry and microelectrode recording of the endplate potentials (EPPs) in the synaptic area of the frog* m. cutaneous pectoris *[11]. The different effects of muscarine in these NMJs could be associated with the heterogeneous localization of mAChRs, in particular, those of M3 subtype: some of these receptors can reside at the nerve ending and be activated by a small amount of the agonist, while the remaining part can be located at some distance from the secretion zone (e.g., on Schwann cells or on the sarcolemma). These remote receptors can be activated at high levels of secretion only or by an exogenous non-hydrolysable agonist such as muscarine or carbachol. The presence of mAChRs on perisynaptic Schwann cells in the frog NMJs is indirectly evidenced by the muscarine- induced increase in intracellular Ca^2+^ ions in this compartment of the neuromuscular contact [30]. The scheme of ACh secretion regulation triggered by M3 subtype mAChRs residing on the sarcolemma was proposed for the lizard motor synapses, involving the synthesis of endocannabinoids, and 2-AG in particular, from muscle membrane lipids [6].

The heterogeneous localization of mAChRs in the NMJ may partly explain the multiple and often ambiguous effects of muscarinic agents on the neuromuscular transmission.

## FUNCTIONAL ROLE OF mAChRs IN SKELETAL MUSCLE

The possibility of autoregulation of ACh release from motor nerve endings on the feedback principle was first shown for nicotinic cholinoreceptors back in the 1960s. The muscarinic regulation was discovered later [4, 5, 31].

Most of the early studies of neuromuscular transmission pointed to the facilitation of neurosecretion upon activation of nicotinic receptors, whereas the muscarinic receptors were believed to play the role of inhibitor of the ACh quantal release [31, 32, 33]. The discovery of different mAChR subtypes (including those that can facilitate neurosecretion) in the innervated areas of skeletal muscles, as well as the ambiguous results of studies performed under different experimental conditions, forced a rethink of this postulate [7, 34, 35, 36]. Thus, it was shown that methoctramine, the blocker of M2/M4 mAChRs, increases the EPP quantal content in the rat NMJ at a physiologically relevant Ca^2+^ level but inhibits the ACh release under reduced Ca^2+^ conditions (or when the amount of ACh in the synaptic cleft is diminished by adding the exogeneous cholinesterase) [36]. One may assume that it is the increased activation of mAChRs, particularly of the M1 and M2 subtypes, that induces the alteration of the EPP quantal content upon the inhibition of synaptic acetylcholinesterase [35]. Although the experiments performed at reduced ambient Ca^2+^ do not unequivocally apportion the physiological role of mAChRs in the synapse, they demonstrate the possibility of switching from one signaling pathway to another and allow one to highlight the effects of the activation of certain mAChRs associated with the alteration of the intracellular Ca^2+^ level (and, therefore, manifest themselves more obviously when the Ca^2+^ level is initially lower).

The difference in the intensity of muscarinic effects on the spontaneous and evoked secretion in frog neuromuscular synapses at reduced and physiologically relevant Ca^2+^ was noted in a number of studies [11, 37]. Under reduced Ca^2+^ conditions, selective blockers of M1, M2/M4, and M3 mAChRs reduced the quantal release of ACh. At a “physiological” Ca^2+^ level, some muscarinic agents influenced the ACh quantal release only at a high-frequency pattern of motor nerve stimulation. Partially, this may happen due to time-delayed processes developing in motor nerve endings upon mAChRs activation. This assumption was indirectly confirmed by the estimation of the Ca^2+^ transient in the nerve ending; that is, the integral signal reflecting the Ca^2+^ metabolism in the cell over a fairly long period of time (several tens of ms) after the action potential arrival. In these experiments, activation of M2 mAChR in frog motor nerve endings led to a small but significant decrease in the amplitude of the Ca^2+^ transient [38].

In addition to the regulation of the amount of ACh secreted from the nerve endings, activation of mAChRs may also lead to changes in the timing of the release process. Along with the EPP quantal content, the timing of transmitter release is a factor ensuring synaptic plasticity [39, 40]. The degree of synchrony of neurosecretion in the NMJ depends on a number of factors such as temperature, the pattern of motor nerve firing, and the presence of physiologically active agents [40, 41, 42]. In frog motor synapses, inactivation of the M2 mAChRs not only modulates the EPP quantal content, but also desynchronizes the ACh release process [11, 43]. Further studies into muscarinic regulation of the timing of ACh secretion were conducted using animals with mutations in the genes encoding different subtypes of mAChRs [44]. M2 mAChR knockout mutants demonstrated, in contrast to wild-type mice, greater sensitivity to the experimental modifications of the Ca^2+^ level in the cytoplasm (variation of [Ca^2+^] in the bathing solution, addition of calcium buffers, etc.). In mutant mice, not only did these manipulations lead to changes in the EPP quantal content, but they also altered the timing of ACh secretion.

M1 receptors may also be involved in controling the timing of ACh secretion [10, 45]. However, in frog NMJs, the involvement of these receptors in the regulation of secretion synchrony was obvious only under conditions of high-frequency stimulation of the motor nerve. The blockade of these mAChRs prevented any increase in the duration of the EPP rise time which, in the case of unchanging temporal parameters of uniquantal EPPs, could be regarded as indirect evidence of a shift in the synchrony of the ACh quanta secretion [10].

The synthesis of positive and negative allosteric modulators of M5 mAChR (compounds VU-023842 and ML-375 [17, 46]) allowed one to better understand the physiological role of M5 mAChRs in skeletal muscles. So, at positive modulation of M5 mAChR, the EPP quantal content and EPP rise time increased, whereas the synaptic depression (serial EPP amplitudes rundown) caused by high-frequency nerve firing was deepened [12].

The effects of mAChR activation or inactivation on motor synapses are not limited to the regulation of the quantal ACh secretion. In the presence of the positive M5 mAChR modulator, compound VU-0238429, the strength of muscle contractions decreased, both during indirect and direct stimulation. This observation supports the postsynaptic localization of M5 mAChRs and the possibility of direct regulation of muscle contractility by ACh [12]. The mechanisms driving such regulation of muscle properties remain unclear. For example, it was shown that the activation of all mAChRs expressed in a mouse fibroblast cell culture (NIH 3T3) can inhibit L-type Ca^2+^ channels via protein kinase C activation [47]. The question of co-localization of M5 receptors with Ca^2+^ channels in skeletal muscles and the possibility of their functional regulation remains open.

It was suggested that activation of M1 mAChR on the sarcolemma by ACh (presumably of non-quantal origin) protects skeletal muscle fibers from early postdenervation depolarization [13]. That is, M1 mAChR can mediate trophic, non-impulse regulation of the resting membrane potential in skeletal muscles. In the absence of nerve stimulation, endogenous activation of the M1 mAChR was detected, modulating the nonquantal release of ACh from the nerve ending, and these receptors apparently resided on muscle fibers; that is, the control of non-quantal secretion could be retrograde [48].

A number of studies have demonstrated the involvement of mAChRs in the structural rearrangements in the synapses. Thus, mAChRs located on the perisynaptic Schwann cell regulate the activity of the glial fibrillary acidic protein (GFAP), which maintains the cell shape and is involved in the regulation of cell proliferation and synaptic plasticity. This regulation is mediated via the alteration of [Ca^2+^]i in perisynaptic Schwann cells. It is assumed that M2 mAChR in the Schwann cells of warm-blooded animals is involved in the control of the proliferation, differentiation, and myelination of these cells [30, 49, 50]. In neuromuscular preparations of newborn rats, muscarinic autoreceptors of the M1, M2 and M4 subtypes can participate in the differentiation of “strong” and “weak” synapses in the case of polyinnervation of muscle fibers at early stages of synaptogenesis [51, 52].

The role of various mAChR subtypes in the maintenance of synaptic stability, growth, and development of mice motor synapses was studied in detail using pharmacological and genetic analysis [7]. Blockade of all five mAChR subtypes with atropine (subcutaneous injections for 7 days) had a pronounced effect, including the disappearance of some nerve endings and the spontaneous sprouting of others, as well as muscle atrophy. Blockade of only M2/M4 mAChR subtypes with methoctramine caused changes at the level of nerve endings, but it did not affect the muscle fibers. Injections of the M3 mAChR blocker 4-DAMP caused complete elimination of nerve endings, but it did not affect the Schwann cells. Similar morphological changes were observed in genetically modified mice: M2-/- mutants were characterized by instability of nerve endings (elimination of nerve terminals accompanied by spontaneous sprouting), while M5 muscarinic receptor knockout mice were characterized by a small size of motor synapses and muscle fiber atrophy.

Thus, different mAChR subtypes ensure the functionality of the tripartite synapse (nerve ending – muscle fiber – Schwann cell) and coordinate the development and morphological properties of the synapse with its activity.

## SIGNALING PATHWAYS ASSOCIATED WITH mAChR ACTIVATION IN THE NMJ

Muscarinic cholinoreceptors can activate numerous signaling pathways in the neuromuscular junction. The classical concept of neuromodulation mediated through odd (M1, M3, M5) and even (M2, M4) mAChR subtypes divides the signaling pathways associated with these subtypes into the activation of Gq and Gi/o proteins. Traditionally, the facilitation of ACh secretion, mediated through the activation of “odd” mAChRs, has been associated with the activation of phospholipase C, leading to the synthesis of inositol-4,5-trisphosphate (IP3) and diacylglycerol (DAG) [53]. IP3 increases the secretion intensity by releasing Ca^2+^ from intracellular stores, and DAG can have a direct effect on the proteins of the exocytotic machinery. Regulation of the activity of protein kinase A through changes in the level of cAMP upon activation of Gi/o proteins leads to the modulation of Ca^2+^ channels, proteins of exocytotic machinery, and also controls the process of ACh loading into vesicles [54, 55, 56, 57]. However, recent studies have shown that in the mouse NMJs, the M1 and M2 mAChR subtypes can use the same targets downstream of G protein activation [58]; that is, there is a reciprocal relationship between M1 and M2 mAChRs, which is implemented through the protein kinase A anchoring protein. Some effects associated with the activation of M2 mAChRs are observed only when the M1 receptors are active (e.g., reduced activity of the catalytic subunits of protein kinase A and elevated activity of regulatory subunits). Other changes may be caused by additional activation of M1 receptors (e.g., an increase in the level of the regulatory protein RIIβ and its release into the cytosol). Moreover, mAChRs may share the same signaling pathways with receptors for other neurotransmitters. For example, it has been shown that in rat NMJs, presynaptic adenosine A2 and muscarinic M1-receptors facilitate neurosecretion, and that these receptors share the same intracellular signaling pathway [59, 60]. Competition between receptors can occur through signal convergence to a common link via the activation of protein kinase A and Ca^2+^ entry through L-type Ca^2+^ channels. Later, it was shown that endogenous adenosine released during rhythmic nerve activity is involved in the fine-tuning of the presynaptic activity of M1 and M2 mAChRs [61]. The prevalence of autofacilitation associated with M1 mAChRs during rhythmic nerve stimulation occurs due to the accumulation of endogenous adenosine in the synapse area, which acts on A1 receptors and attenuates the effects associated with the activation of the M2 mAChRs. A similar phenomenon — the absence of any effect of M2 mAChR activation on spontaneous ACh secretion in frog synapses in response to the action of adenosine — was observed in frog synapses [62].

In the lizard NMJs, mAChR activation led to a two-phase modulation of neurosecretion: short-term ( < 12 min) activation of M3 receptors by muscarine reduced the quantal release of ACh, while the longer term activation of M1 receptors, on the contrary, increased it, and both of these effects depended on the level of nitric oxide in the synaptic contact area [63]. The severity of the effects associated with the stimulation of M1 receptors was dependent on the cAMP level and protein kinase A activity. The decline in EPP quantal content upon activation of M3 receptors residing on the muscle cell is mediated via the rise of the synthesis of endocannabinoids, probably 2-AG [6]. In the synaptic cleft, endocannabinoids bind to the CB1-type receptors on presynaptic nerve endings, thus restricting Ca^2+^ entry and leading to a decrease in ACh. Moreover, at least one link in this regulatory chain requires the production of nitric oxide (either in muscles or in Schwann cells).

It is worth noting that in the study performed in frog NMJs [64], activation of M3 mAChRs also reduced the EPP quantal content; however, this suppression of ACh secretion was associated solely with the activation of NO synthase and an increase in the nitric oxide level: it did not involve endocannabinoid production. This fact, however, does not rule out the presence and functional role of CB1 type cannabinoid receptors in the motor synapses of frog. The activity of NO synthase may be elevated due to an increase in the [Ca^2+^]i upon activation of the Gq proteins associated with the M3 mAChR subtype. It is interesting to note that inhibition of phosphoinositide 3-kinase (PI3K) by wortmannin prevented the restoration of the original level of secretion after muscarine had been removed from the bathing solution; that is, the application of muscarine led to an imbalance between the synthesis of membrane phospholipids and their breakdown, which apparently could affect the properties of a number of signaling molecules associated with membranes and involved in the regulation of exocytosis.

Another mechanism of muscarinic regulation of ACh secretion in motor synapses is associated with the activity of G-protein-gated K+ channels (GIRK channels). The activation of GIRK channels by Gi proteins usually leads to hyperpolarization of the cell and reduces its excitability. One of the metabotropic receptors coupled to Gi proteins is the M2 mAChR subtype. Studies involving the fluorescent label FluxOR™ made it possible to visualize the opening of K+ channels upon activation of M2 mAChR in frog skeletal muscle [65]. These experiments directly showed that GIRK channels are functionally active in frog motor synapses and that they are coupled to M2 mAChRs. An analysis of EPPs and MEPPs recorded in the presence of an activator (ML-297) and a blocker (tertiapin- Q) of GIRK channels has revealed that these channels demonstrate an ambivalent behavior in frog NMJs. Depending on the level of extracellular Ca^2+^, M2 mAChRs can either inhibit or increase the level of evoked ACh secretion in frog NMJs [11]. One can conjecture that extracellular Ca^2+^ can serve as a switch between the stimulatory and inhibitory functions of M2 mAChRs; that is, that these receptors can both activate and inhibit GIRK channels, differentially modulating the evoked neurotransmitter release. The next link in this signaling circuit downstream the GIRK channel is the L-type Ca^2+^ channel. It is suggested that the suppression (due to hyperpolarization) of the asynchronous (spontaneous) activity of these Ca^2+^ channels during interstimulus intervals can ensure that they successfully fire in response to the action potential.

Another signaling pathway associated with M2 mAChRs in the NMJs of both cold-blooded and warm-blooded animals is associated with the tonic block of the exocytotic machinery upon activation of these receptors and its elimination upon depolarization of the motor nerve ending [66]. It has been suggested that at rest, M2 mAChRs have an increased affinity for ACh and, when activated, switch the exocytotic machinery to the state of tonic block. When the presynaptic membrane is depolarized, the affinity of M2 mAChRs for ACh decreases, the ACh molecules dissociate, and the proteins of the exocytotic machinery can then interact with Ca^2+^, which ultimately leads to ACh release. Later, the dependence of the ACh dissociation constant on the resting membrane potential for M2 mAChRs was demonstrated directly on oocytes by K+ currents recording through GIRK channels and by assessing the degree of binding and unbinding of labeled ACh [67]. Using the “uncaged” carbachol, it was shown that in the case of rapid (within several ms) release of cholinomimetic in the area of a motor synapse, blockade of M2 mAChRs leads to a significant, dose-dependent decrease in the quantal release of ACh in wild-type mice, while in M2 (-/-) mutants carbachol has no effect on the intensity of secretion. This can be interpreted as the involvement of M2 mAChRs in the earliest phase of secretion, within a few ms after depolarization of the nerve ending [68].

In rat Schwann cells, M2 receptors, in addition to the canonical pathway associated with the Gi protein, also activate non-canonical pathways, including the PI3K/AKT/mTOR signaling pathway, which can modulate the proliferation and migration of these glial cells [50].

## COMPARISON OF SIGNALING PATHWAYS ASSOCIATED WITH mAChR ACTIVATION AT CENTRAL AND MOTOR SYNAPSES

As a part of this review, it was interesting to compare some signaling pathways associated with mAChRs in motor and central synapses.

In the CNS, mAChRs are located in various brain regions innervated by cholinergic neurons, both on postsynaptic and presynaptic membranes, as well as in glial cells. mAChRs are involved in a variety of processes such as learning, concentration of attention, regulation of sleep-wake cycle, motor control, and others. mAChR activation is associated with effects such as postsynaptic excitation, postsynaptic inhibition, and presynaptic autoinhibition [69, 70].

One of the mechanisms responsible for postsynaptic excitation is the inhibition of the voltage-gated K+ channels (M-channels) associated with M1/M3/M5 subtypes, as a result of the activation of phospholipase C. M channels include some members of the Kv7 subfamily: mainly Kv7.2 and Kv7.3 [71, 72]. To stabilize them in the open state, a given density of phosphatidylinositol 4, 5-bisphosphate (PIP2) in the cell membrane is required [73, 74]. Rapid hydrolysis of PIP2 by phospholipase C leads to the inactivation of the K+ channel, which causes depolarization and enhanced excitability of the cell. This mechanism was first encountered in sympathetic neurons, but it is also typical of some central neurons (e.g., hippocampal pyramidal neurons, cortical pyramidal neurons). M channels are usually concentrated in the axon’s initial segment, where they control the action potential threshold. An additional excitation mechanism associated with the activation of odd-numbered mAChRs and depletion of membrane lipids is the inhibition of some other K+ channels; for example, Ca^2+^-dependent K+ channels or leak K+ channels [75, 76, 77, 78].

M channels (Kv7.2, Kv7.3 and Kv7.4) were found in striated muscles [79, 80]; they are credited with the role of regulators of skeletal muscle differentiation and maintenance of muscle tone [81, 82, 83]. Considering the presence of odd-numbered mAChRs on the sarcolemma, their possible co-localization with Kv7 channels and modulation of K+ channels functioning seems a very plausible idea that could explain some of the effects of muscarinic agents on muscles.

Postsynaptic inhibition occurs in the central nervous system as a result of the activation of inward rectifying K+ channels (GIRK channels) associated with the M2 mAChRs. This mechanism was first discovered in sympathetic and parasympathetic neurons [84], and later similar M2-mediated effects of ACh were detected in some central neurons [70, 85, 86, 87]. The slow inhibitory postsynaptic potential is a delayed hyperpolarization starting approximately 50 ms after the “nicotinic” EPP. This hyperpolarization closely resembles the myocardial response to vagal stimulation and results from the activation of the Gi-protein K+ inward rectifier channels (mainly Kir3.1 and Kir3.2) following the activation of the M2 mAChRs. Coupling of M2 mAChRs with GIRK channels was shown for the neuromuscular synapse [65]. In motor synapses, GIRK channels are localized on the presynaptic membrane; it turns out that they can not only reduce cell excitability, but, under certain conditions, also facilitate ACh secretion due to the suppression of “calcium noise” during rest intervals. At vertebrate motor synapses, this signaling pathway is involved in the autoregulation of neurosecretion**.**

As for autoregulation in the CNS, here presynaptic inhibition (autoinhibition) is typically associated with direct inactivation of voltage-dependent Ca^2+^ channels coupled with M2/M4 receptors. In sympathetic neurons, these two mAChR subtypes and their related Gi and Go proteins and effector channels can form the functional microdomains or signalosomes [88], possibly with the participation of some auxiliary proteins [89].

Every year, new mechanisms responsible for signal transmission via metabotropic receptors are being discovered, and the already known ones are becoming more complex due to the identification of additional isoforms of the molecules involved in signal transmission, the recognition of new points of intersection of signaling pathways, and the identification of differences in signal transmission specific to different cells. It is traditionally believed that odd receptor subtypes (M1, M3 and M5) activate phospholipase C through pertussis toxin-insensitive G-proteins of the Gq family, and that receptors of the M2 and M4 subtypes regulate the activity of adenylate cyclase (using pertussis toxin-sensitive G-proteins of the Gi family) without PLC stimulation. However, this specificity is not absolute and “even” mAChRs can activate the α-subunit of the Gs and Gq/11 proteins, thus triggering numerous signaling pathways, depending on the nature and concentrations of the agonist [90, 91, 92].

## NEUROMUSCULAR PATHOLOGIES ASSOCIATED WITH IMPAIRED MUSCARINIC REGULATION

An imbalance in the cholinergic system is the major cause behind the symptoms in many neurological diseases, including Alzheimer’s and Parkinson’s, schizophrenia, depression, and bipolar disorder [16]. However, there is currently scant data directly connecting any diseases with defects in the muscarinic regulation of skeletal muscles, and neuromuscular transmission in particular.

Violation of muscarinic adaptation may be one of the pathogenetic factors that lead to the development of amyotrophic lateral sclerosis. It is known that perisynaptic Schwann cells are involved in maintaining the stability and normal functioning of motor synapses, and that mAChRs play an important role in the implementation of these processes. One of the functions of Schwann cells is rapid removal of axonal debris after damage to peripheral nerve fibers [93]. An increase in the phagocytic activity of Schwann cells is associated with the expression of galectin-3, and the level of mAChR activation is a determining factor when a Schwann cell switches from the maintenance mode to the repair mode [94]. A mouse model of amyotrophic lateral sclerosis (SOD1 strain) exhibits increased mAChR activation in Schwann cells during the pre-onset stage of the disease [95] and an inability to activate galectin-3 during nerve injury [96, 97].

In patients with chronic fatigue syndrome (myalgic encephalomyelitis) and Lambert–Eaton myasthenic syndrome, the enhanced production of autoantibodies to certain mAChR subtypes (M1, M3, M4) was detected, which is likely to aggravate the severity of some symptoms of these diseases, manifested as impaired motor activity [98, 99].

## CONCLUSION

**Fig. 1:**
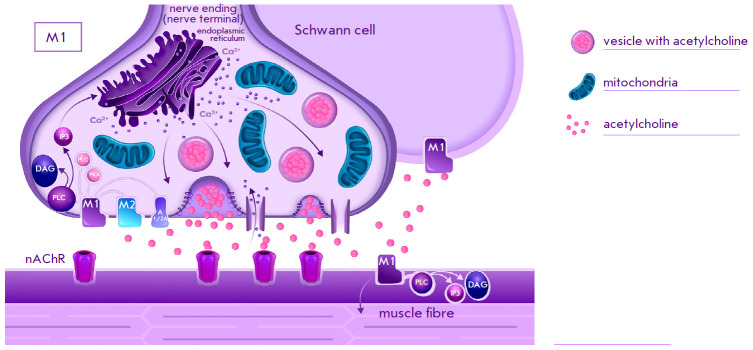
Schematic representation of the localization of M1 subtype muscarinic acetylcholine receptors and the associated signaling pathways in the neuromuscular synapse of vertebrates [7, 8, 29, 48, 52, 58]; nAChR, nicotinic acetylcholine receptor; PLC, phospholipase C; DAG, diacylglycerol; IP3, inositol triphosphate; PKC, protein kinase C; PKA, protein kinase A; A1/2A, adenosine receptor

**Fig. 2:**
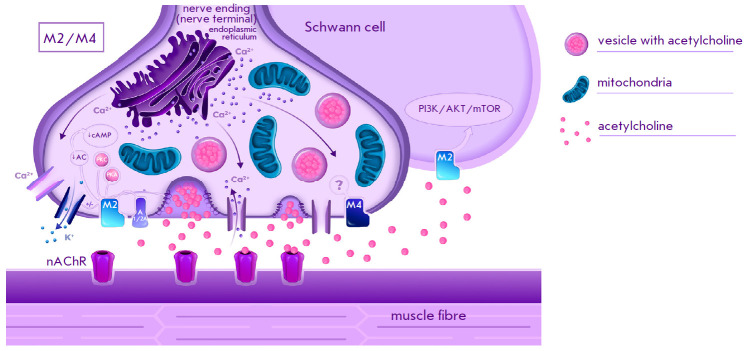
Schematic representation of the localization of M2 and M4 subtypes muscarinic acetylcholine receptors and the associated signaling pathways in the neuromuscular synapse of vertebrates [7, 8, 29, 48, 52, 58]; nAChR, nicotinic acetylcholine receptor; AC, adenylate cyclase; cAMP, cyclic adenosine monophosphate; PKC, protein kinase C; PKA, protein kinase A; A1/2A, adenosine receptor; PI3K/AKT/mTOR – the signaling pathway involving phosphatidylinositol 3-kinase, protein kinase B, and mTOR kinase

**Fig. 3:**
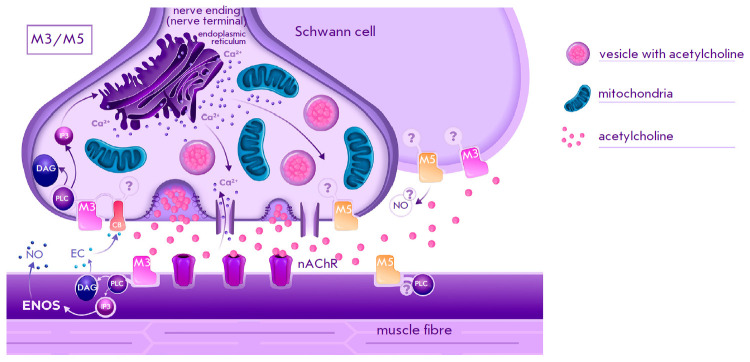
Schematic representation of the localization of M3 and M5 subtypes muscarinic acetylcholine receptors and the associated signaling pathways in the neuromuscular synapse of vertebrates [7, 8, 29, 48, 52, 58]; nChR, nicotinic acetylcholine receptor, PLC, phospholipase C, DAG, diacylglycerol, IP3, inositol triphosphate, NO, nitric oxide, ENOS, endothelial form of NO synthase, EC, endocannabinoid (presumably 2-AG), CB, endocannabinoid receptor

This review has attempted to summarize the currently known facts and hypotheses as they relate to the functions of muscarinic receptors in the skeletal muscles of cold- and warm-blooded animals. Basic information and assumptions about the localization, consequences of pharmacological and genetic influences and mAChR-related signaling cascades in the NMJ and vertebrate skeletal muscle are presented in *Table* and *Figure 1*, *Figure 2*, *Figure 3*.

Today, there is no doubt that all five (M1–M5) currently known mAChR subtypes are present in vertebrate skeletal muscles. The signaling pathways associated with the activation of various mAChR subtypes in vertebrate skeletal muscles are diverse, and the effects of the activation of these receptors vary in duration (from several ms to tens of minutes) and, apparently, retain the possibility of “switching” from one signaling pathway to another depending on factors of internal or external nature. Some of these intracellular mechanisms are associated, in one way or another, with changes in the level of intracellular Ca^2+^ (by regulating its release from intracellular stores or modifying the functions of Ca^2+^ channels). Other possible signaling pathways involve a direct effect on the exocytotic machinery; for example, through the regulation of protein kinase A activity, phosphorylation of the SNAP-25 protein, etc.

The functions of mAChRs in skeletal muscle are not limited to the autoregulation of ACh secretion. Muscarinic receptors of the M1 and M2 subtypes can be involved in the regulation of the timing of ACh release. Odd-numbered mAChRs can be located on the sarcolemma and regulate the contractility of muscle fibers or participate in the maintenance of the resting membrane potential.

It is worth noting that mAChRs *per se *are the targets of various endogenous factors, such as free radicals [100, 101]. In addition, they are voltage-sensitive (moreover, in the physiological range of shifts in the cell membrane potential) [102, 103, 104]. Therefore, we can envisage the possibility of a dynamic regulation of mAChRs properties at different patterns of synapse operation (e.g., reduction of the probability of activation during the generation of an action potential or when the NMJ operates in a high-frequency mode).

ligands, including allosteric modulators, are actively used for treating various pathologies, and a targeted search for novel, highly selective muscarinic agents as potential therapeutic agents is currently underway [1, 2, 16]. The localization of all currently known mAChR subtypes in skeletal muscle and the diversity of the signaling cascades associated with their activation should be taken into account when using muscarinic agents as medications.

**Table 1:** Localization and functions of mAChRs in vertebrate skeletal muscles

Subtype	Localization	Effects associated with activation	Putative signaling pathways
M1	nerve ending [7, 11, 29]; sarcolemma [8]; Schwann cell [29]	augmentation of EPP quantal content [10, 11, 29, 31, 63]; regulation of non-quantal ACh release and muscle resting membrane potential [8, 13]; differentiation of “strong” and “weak” synapses at the early stages of synaptogenesis [51, 52]	activation of phospholipase C, [Ca^2+^]i elevation, regulation of the activities of protein kinases A and C [52, 58, 63]
M2	nerve ending, Schwann cell [7, 29, 30, 49, 50]	Ca^2+^-dependent regulation of EPP quantal content and the timing of ACh release [11, 29, 34, 43, 44, 65, 66]; regulation of Schwann cell differentiation and proliferation [30, 49, 50]; control of the development of motor nerve endings during the ontogenesis [7], differentiation of “strong” and “weak” synapses at the early stages of synaptogenesis [51, 52]	regulation of cAMP level and protein kinases A and C activities [52, 58]; tonic block of the exocytosis apparatus [66]; Ca^2+^-dependent regulation of K+ channel (GIRK) and L-type Ca^2+^ channel [65]; PI3K/ AKT/mTOR signaling pathway [50]
M3	nerve ending [29]; sarcolemma [63]; Schwann cell [29]	activation of phospholipase C, elevation of endocannabinoids and nitric oxide production [6, 63, 64]	activation of phospholipase C, elevation of endocannabinoids and nitric oxide production [6, 63, 64]
M4	nerve ending [7, 29]; Schwann cell [29]	regulation of EPP quantal content [6]; differentiation of “strong” and “weak” synapses at the early stages of synaptogenesis [51, 52]	no data
M5	sarcolemma [7]; nerve ending? Schwann cell?	control of muscle growth and synaptic contact formation during the ontogenesis [7], regulation of muscle contractility [12]; augmentation of EPP quantal content [12]	no data

## Abbreviations

ACh: acetylcholine
mAChR: muscarinic cholinoreceptor
NMJ: neuromuscular junction
EPP: endplate potential
